# Oncogenic cell fate decision in breast epithelial cells I: growth factors and mechanisms

**DOI:** 10.21203/rs.3.rs-9365348/v1

**Published:** 2026-05-22

**Authors:** Divya Murali, Shakti Gupta, Shankar Subramaniam

**Affiliations:** a Department of Bioengineering, UC San Diego; b Departments of Computer Science & Engineering, Cellular & Molecular Medicine, and Nanoengineering, UC San Diego

**Keywords:** Breast cancer, multiomics, systems biology, growth factors, hormone receptors

## Abstract

**Background:**

Breast cancer cell heterogeneity and cellular fate are governed by a variety of molecular mechanisms. The LINCS (Library of Integrated Network-based Cellular Signatures) consortium performed multi-omics experiments on normal breast epithelial cells, MCF10a, to deduce temporal mechanisms of regulation and cell state signatures contributing to pro-oncogenic phenotypes.

**Methods:**

Normal breast epithelial cells were treated with oncogenic ligands such as EGF, HGF, and OSM, and multi-modal measurements including Reverse phase protein Assay (RPPA), RNA-seq, ATAC-seq, and Cyclic immune-fluorescence (Cyclic-IF) were performed.

**Result:**

In our integrated analyses of the data to elucidate mechanisms, contextual functional networks were constructed by integrating protein signaling, transcription factor activity, and gene expression. Phenotypic changes in response to the ligands consisted of cell cycle modifications leading to oncogenic events such as loss of apoptosis and induction of EMT (Epithelial to Mesenchymal Transition). Activation of mTOR was observed with all ligands, which led to the activation of E2F1. Downstream transcriptomic regulation of E2F1 led to an increase in both oncogenic signaling and EMT. Additionally, under OSM treatment, activation of STAT3 facilitated the enhancement of EMT via transcriptomic regulation of JUN and FOS. These findings were further validated using the chromatin changes seen in ATAC-seq and protein localization as seen in Cyclic-IF assay.

**Conclusion:**

This analysis provides valuable insights into the mechanisms of transcriptional regulation during oncogenesis in normal breast cells treated with growth factors and can aid in the discovery of novel drug targets and treatments

## Introduction:

Breast cancer is heterogeneous in nature and is classified based on the expression of hormone receptors (HR) and HER2 including Luminal A (HER2−, HR+), luminal B (HER2+, HR+), HER2 overexpressing (HER2+, HR-), and triple-negative breast cancer (HER2-, HR-)[1–4]. Cellular heterogeneity arises from underlying cellular and molecular landscape, involving protein interactions, gene expression, and changes in the epigenetic landscape, leading to diverse tumor phenotypes[5, 6], which include increased proliferation, angiogenesis, epithelial to mesenchymal transition (EMT), metastasis, cell plasticity, and loss of apoptosis. These processes help in tumor initiation, leading to changes in cell state, invasion, and proliferation. Integrative analyses of cellular signaling, transcriptomic, and epigenomic data provide the opportunity to identify mechanisms that induce and promote tumor pathology.

Tumor growth and proliferation are accompanied by an increase in both autocrine and paracrine signaling of growth regulators[7–10]. In general, growth factors are responsible for proliferation, development, and homeostasis in normal breast cells. Aberrant expression of growth factor proteins and abnormal cellular response to these proteins lead to oncogenesis. Since the discovery of soluble growth factor EGF[11], studies have established that amplification or overexpression of endogenous growth factors such as EGF, HGF, TGFβ, VEGF, and IGF leads to cell proliferation, migration, angiogenesis, and metastasis[10, 12–15]. While EGF is required for the growth and development of breast epithelial cells, overexpression of EGF leads to a sustained activation of several crucial members of the ERBB Receptor Tyrosine Kinases (RTKs) family. Sustained activation of these RTKs is known to mediate MAPK mediated tumorigenesis, c-MYC mediated anti-apoptotic signals, hyperactivation, and nuclear localization of kinases such as p-AKT and p-ERK1/2 which promote proliferation and EMT[16, 17]. Human secreted cytokine Oncostatin-M (OSM), a growth regulator of the IL-6 superfamily, is also a potent inducer of cancer cell de-differentiation and stem cell plasticity[18]. OSM also causes the induction of transcription factors such as SNAI1, SNAI2, ZEB1, FN1, and CDH2 which are well-known markers of EMT in human breast cancer cells[16].

High throughput sequencing studies in loss/gain of function using siRNA, shRNA, and CRISPR have led to new avenues for understanding the genetic vulnerabilities and discovery of drug targets[17, 19, 20]. These advances have led to the identification and characterization of various oncogenes such as growth factor receptors (ERBB2/3, c-MET, OSMR), cell cycle modulators (CDKs, CCNDs, and others), kinases (AKT, PI3K), and transcription factors (c-MYC, c-FOS). For example, loss of function of ERBB2/3 blocks cell proliferation and cell cycle[21, 22] and inhibition of cell cycle regulators such as CDKs (1–6) induces apoptosis[23–26]. Upregulation of PIK3CA leads to the formation of malignant tumors[27]. The oncogenic role of PIK3CA and CDK proteins are attributed to ZEB1 activation which controls EMT[28]. OSMR also signals via JAK/STAT pathway where STAT3 regulates cyclin D-1 and c-MYC to increase proliferation. STAT3 can also inhibit BAX/BCL-2-related caspase-dependent apoptosis[29–31].

Recent developments in high throughput technologies have aided in early diagnosis and development of novel therapeutic strategies[32–38]. In order to provide an integrated view of cellular mechanisms that lead to the development of tumor, the National Institutes of Health launched a multi-institutional study, called Library of Integrated Network-based Cellular Signatures (LINCS), which made multi-omics measurements on normal breast epithelial cells, MCF10a treated with tumor growth-regulating ligands namely EGF, HGF, and OSM[39]. Experimental measurements were taken longitudinally with reverse phase protein assay (RPPA), RNA-seq, ATAC-seq and cyclic-IF. These measurements provided the ability to decipher potential tumor activating and suppressing mechanisms through integrative analyses. In our analysis, integration of multi-modal data showed that EGF, HGF, and OSM treated cells activate mTOR, STAT3, and ERK pathways leading to enhanced cell cycle, inflammation, and EMT. These phenotypes are transcriptionally regulated by E2F1 in all three ligands and are further enhanced in OSM treated cells by the activation of the AP1 complex. Our study offers key insights into pathways and underlying mechanisms for oncogenesis in normal breast cells to help identify early-stage mechanisms and potential therapeutic targets. We present the results of these analyses in the following sections.

## Methods:

### Data:

NIH LINCS program contributed to the creation and preprocessing of an MCF10a perturbation dataset at Oregon Health and Science University (OHSU). MCF10a cells were treated with ligands as follows: 10 ng/ml EGF, 40 ng/ml HGF, and 10 ng/ml OSM. Samples were collected at 1, 4, 8, 24 and 48 hours post treatment for RPPA and cyclic-IF measurements, and 24 and 48 hours for RNA-seq and ATAC-Seq.

### RPPA data analysis

RPPA protein samples were prepared and preprocessed according to the standard preprocessing methods developed at the MD Anderson Cancer Center RPPA core and passed QC checks. The normalized RPPA log2 values are joined with their experimental metadata obtained from the MD Anderson cancer center and stored as level 3 data. Replicates were median summarized in the RPPA Level-4 data and obtained from Synapse for 1, 4, 8, 24, and 48-hour time points for treatment versus PBS control. We used a log_2_fold change (FC) cutoff for downstream functional analysis. |FC| >1.3

### RNA-seq data processing

RNA-seq reads were mapped to the GRCh38.99 human transcriptome using OMICSOFT. The resulting count matrix normalization and differential analysis was performed using the “DEseq2” package available through R/Bioconductor[40]. Genes with at least one count, were retained for further analysis within each matrix. The pairwise comparisons for each treatment at each timepoint were performed with respect to their respective PBS controls at every timepoint. DEGs were called at a Benjamini-Hochberg false adjusted p < 0.05.

### ATAC-seq data processing

ATAC-seq data preprocessing was performed using TrimGalore! package, removing sequencing adaptors and selecting for all paired-end sequences. Trimmed reads were aligned to the GRCh38 human genome using BBMap v37.95 in the BBTools suite followed by sorting and indexing of bam files using SAMtools v1.3, and annotation of PCR duplicates using the Picard v2.3.0 MarkDuplicates function. All duplicates and mitochondrial, chromosome X, chromosome Y, and Epstein-Barr virus (EBV) reads were removed using SAMtools v1.3. Only seventeen different samples were suitable for further analysis. MACS2 was used to call peaks on the ATAC-seq data and determine regions of open chromatin. The specifically called open regions of chromatin were then passed to the R package Diffbind v2.8.0 to determine regions of differential accessibility between each control and treatment conditions using DESEQ2. Differentially accessible regions of DNA were annotated using the R package, ChIPseeker, defining the promoter region −1000 to 500 bp from the TSS. Genes with differential chromatin expression were defined as those called DEGs (|FC| > 1.3 and FDR P-adjusted p-value < 0.05) as described above and had a differentially accessible peak (passing the threshold of |FC| > 1.3 and adjusted p-value FDR P < 0.05) occurring in a promoter. ATAC-seq enrichment analysis was conducted to determine the TF footprints associated with the gain or loss of chromatin accessibility and motifs enriched for these regions using GimmeMotifs. The gimmemotifs function was used with both the HOCOMOCOv11 pwm databases and the Homer motif-finding algorithm.

### Enrichment analysis and Transcriptional regulatory network:

Pathway enrichment analysis was identified using hypergeometric enrichment of DEGs in KEGG, Gene ontology, Hallmark and Reactome databases. Functional pathways describing temporal activation were constructed through a combination of curated pathways and manual literature search. Enrichment terms ranked by their p-value are presented for any comparative analysis.

Using the TRANSFAC database (version 2020), TFs-target relationships were identified, and TFs were enriched using hypergeometric enrichment analysis using an R package. The identified transcriptional networks, including TFs and their targets, were further filtered based on whether they were significantly differentially expressed genes (DEGs) in treatment vs. control at 24 and 48 hours, respectively.

### Identification of ligand specific gene expression changes

Identification of ligand-specific gene expression by pairwise comparison across ligand treatments was performed for all combinations of three treatments and controls to find differentially regulated genes between any two compared conditions. To find the ‘ligand specific’ outcomes for a condition, the intersection of three pairwise comparisons, obtained between the condition and all other conditions, was taken. This cross intersection allowed us to remove the commonly regulated genes. For example, to find EGF-specific genes, we first applied the DESEQ2 between EGF and control (PBS), giving a list of genes differentially regulated in EGF versus PBS. Then the same conditions were applied between EGF and OSM; EGF and HGF respectively, each of which returned a list of genes differentially regulated in EGF versus the compared condition. p-value cutoff ≤0.05 was used for all the cases. The intersection of these lists represented the genes differentially regulated in EGF compared with all other conditions. We denoted this intersection as EGF-specific genes. Similarly, this method was repeated for HGF, and OSM, revealing the ligand specific genes for each condition.

## Results

The cell fate of epithelial cells to maintain homeostasis or transform to tumor cells results from a dynamic interplay between cellular mechanisms involving signaling and growth factors[41]. In this project, MCF10a cells was treated with growth-regulatory ligands EGF, HGF and OSM to assess mechanisms involved in the cell fate processes. Alterations in proteins were measured at 1, 4, 8, 24, and 48 hours post treatment using reverse phase protein array (RPPA), alterations in transcripts and chromatin accessibility measured at 24 and 48 hours post treatment using RNA-seq and ATAC-seq methods, and protein expression and localization were monitored at 1, 4, 8, 24, and 48 hours post treatment using cyclic-immunofluorescence (cyclic-IF) method (Fig. S1).

### Growth regulatory signaling mechanisms.

RPPA measurements showed significant overlap in response to the three treatments (EGF, HGF, and OSM), with alteration at least at one time point for 95 proteins with EGF, 93 with HGF, and 89 with OSM treatments with respect to PBS control (Fig. 1a). RPPA characterization of canonically defined oncoproteins revealed changes in expression of 44 oncoproteins[42–44] (Fig. 1c). Analysis of all significant proteins at each individual time point revealed consistent functional enrichment of several modules across all treatments. The functional enrichment included an increase in hallmark oncogenic phenotypes such as cell growth and proliferation (cell cycle, mTOR signaling, PI3K signaling, MAPK signaling, cellular senescence, TGFβ signaling), inflammation (HIF1α signaling, TNFα signaling), angiogenesis (AMPK signaling, VEGF signaling, ERK signaling), and cytoskeletal changes (gap junctions, tight junctions) (Fig. 1b (i–iii)). Additionally, OSM treatment showed an increase in EMT processes (Hippo signaling, VEGF, angiogenesis) and pro-inflammatory processes (chemokine signaling, IL17 differentiation).

All three treatments activated Receptor tyrosine kinases (RTKs) (HER2_pY1248 and HER3_pY1289) that can lead to the activation of PI3K/mTOR, MEK/ERK, and STAT3 signaling pathways (Fig.1d). However, EGF showed a downregulation of both EGFR and ERBB2 total proteins. Similar activation of AKT, ERK and mTOR was observed as early as 1 hour, along with an increase in activation of its targets EIF4EBP1, S6K at later time points as measured by RPPA in all three treatments. An increase in mTOR1 via PIK3CA led to the increase in the protein expression of TP53BP1, PXN, FOXM1, EIF4EBP1, RPS6, and FOXO3. PIK3CA is also known to the increase phosphorylation of AKT and S6 which play a significant role in proliferation, angiogenesis, metabolism, and loss of apoptosis. Further inactivation of CDKN1A, CDKN1B was observed in all three treatments, indicating cell cycle progression. However, although proteins such as AKT, STAT3 and GSK3B showed consistent trends in all three treatments, EGF showed a significant increase in the expression of phosphorylated AKT-pS473/pS308, ERBB2-pY1248 and GSK3A-pS21-S9 compared the other treatments, indicating treatment-specific activation of downstream pathways. Additionally, in OSM treatment, phosphorylation of STAT3-pY705 led to its nuclear localization as seen in the cyclicIF measurements and triggered enhanced phenotypic changes in cell cycle (CCND2, MYC), anti-apoptosis (BCL2), EMT (SNAIL, ZEB1, HIF1A), and Metastasis (SERPINE, MYC)[45, 46]. HIF1A further upregulated VEGF and ZEB1, thus, promoting EMT, migration, and invasion.

### Proteo-Transcriptomic regulation of cell fate - oncogenesis and EMT.

**Fig 2:**
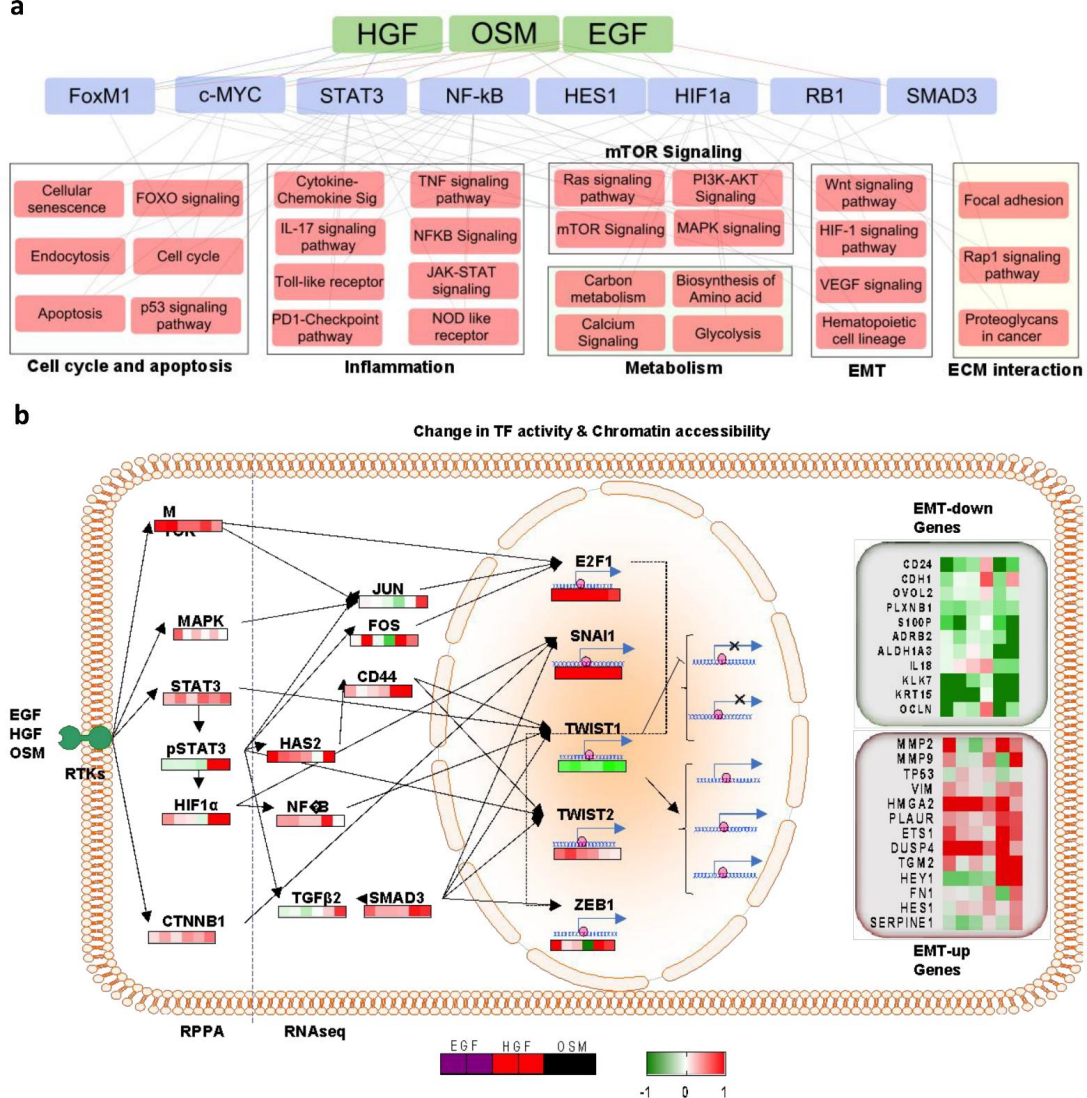
Common oncogenic signaling for EGF, HGF, and OSM treatments from proteomic and transcriptomic analyses. a) KEGG Functional enrichment modules of RPPA TFs for all three ligand treatments and their transcriptional targets show activation of the cell cycle, mTOR, ECM interaction, inflammation, and EMT via SMAD3, HIF1A, STAT3, NFKB, RB1, HES1, c-MYC and FOXM1. b) A schematic model of EMT signaling in EGF, HGF, and OSM treatments showing activation of EMT TFs, namely, E2F1, SNAI1 via SMAD3, CTNNB1, TWIST2 via STAT3, CD44 and SMAD3 and HIF1α, and ZEB1 via SMAD3 and E2F1. mTOR downstream genes JUN, and FOS lead to the activation of the AP1 complex which further leads to the regulation of EMT genes. FC heatmaps for the significant proteins at 24 and 48-hour time points (EGF, HGF, and OSM are represented pairwise in sequence.)

RPPA data showed the upregulation of several transcription factors (TF). To further characterize the role of RPPA-TFs in altering transcriptional regulation, we performed an analysis of the RPPA-TFs and their differentially expressed (DE) target genes obtained from RNA-seq analysis. Functional enrichment was performed for the targets of each TF individually. Analysis showed common modules such as cell cycle, ECM interaction, mTOR signaling pathway, metabolism, inflammation, and EMT across multiple TFs and downregulation of cellular senescence, endocytosis, and p53 mediated apoptosis (Fig 2a).

EMT causes dissociation of cells, loss of polarity, and an increase in the plasticity of cells[47–50]. EMT-related processes such as HIF1α signaling, proteoglycans in cancer, RAP signaling were enriched in the above RPPA-TF-target analysis. Canonical markers of EMT[51–53] such as CDH2, VIM, SMN1, CTNNB1, and FN1 were significantly upregulated and epithelial markers such as CDH1, OCLN and CD24 were downregulated in all three treatments (Fig. 2b). All three ligands showed induction of EMT via mTOR, MAPK, and ERK pathways. These pathways regulate E2F1 which acts as an EMT regulator directly via mTOR and indirectly via ZEB1/2. E2F1 also upregulated MMPs and ADAM12 which further upregulated EMT[54]. In OSM, the transcriptomic regulation of HIF1α, TGFRβ1, JUN, and FOS via STAT3 led to the further activation of TFs (SNAI1and ZEB1) which regulate EMT gene expression[16, 55–58]

### Transcriptional regulation and cellular phenotypes

**Fig3:**
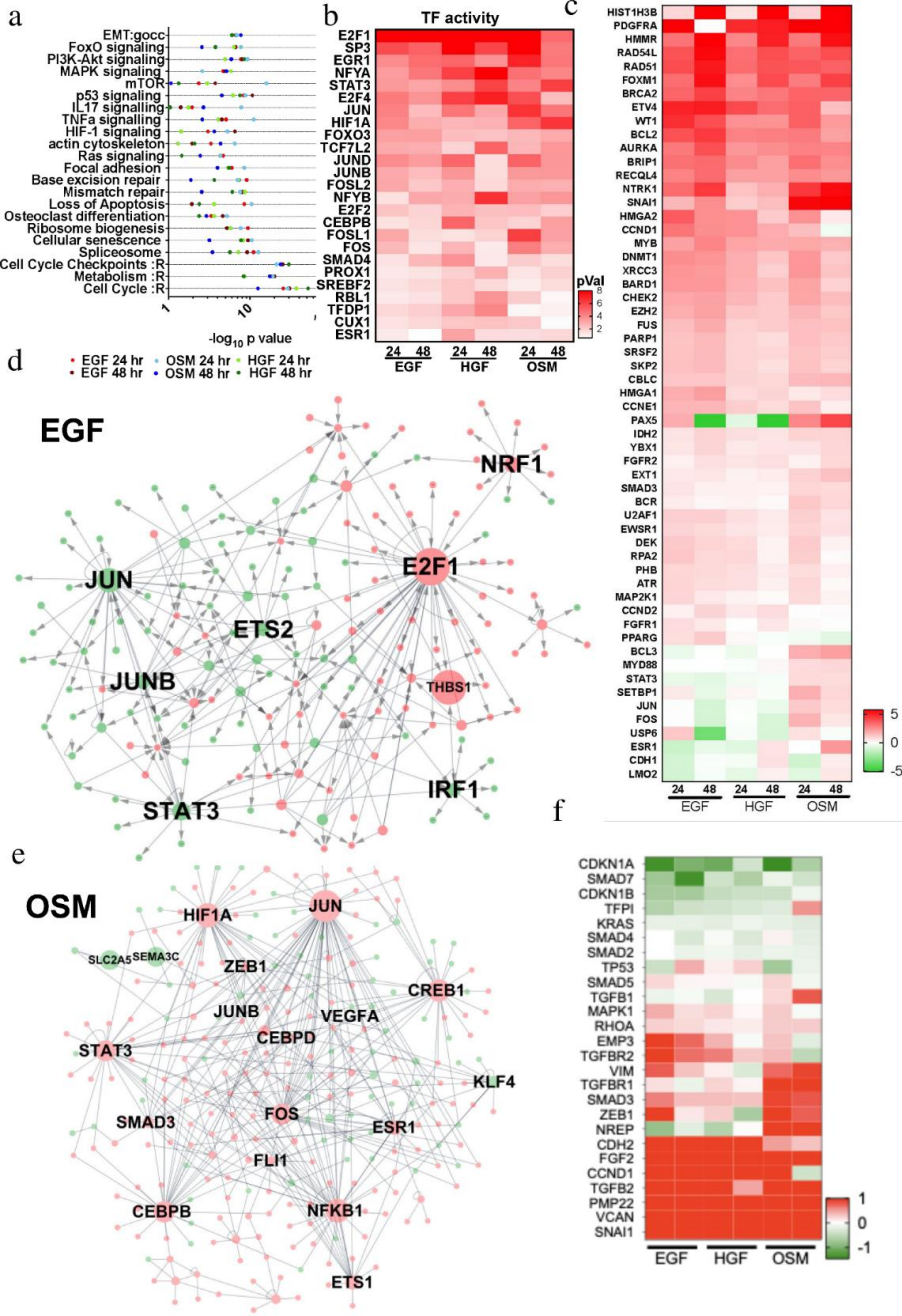
Transcriptomic regulation of oncogenesis a) Pathway enrichment analysis of gene expression signatures using the GO-BP, Hallmark, and Reactome database for the EGF, HGF, and OSM treatments. Dot color represents the treatment at 24, and 48 hours. b) Hypergeometric enrichment of common DE TF across the three treatments based on known DE targets using TRANSFAC database for TFs regulating genes at 24 and 48 hours. c) Fold change heat map of DE oncogene expression at 24 and 48- hours from transcriptomic data. Genes show an increase in the expression of oncogenesis. d)TF target network using DE for EGF-specific Treatment. E2F1 shows an enhanced effect in EGF-specific treatment. e) TF target network using DE genes for OSM-specific treatment. STAT3, JUN, FOS, and HIF1α play important roles in OSM treatment. f) Fold change heat map of genes involved in TGFβ pathway at 24 and 48 hours from transcriptomic data. (DE genes: p-value<0.05)

To evaluate overall transcriptional changes that contribute to downstream functional changes, we analyzed all DE genes in EGF, HGF, and OSM treatments at 24 and 48 hours (Fig. S2). Functional enrichment showed cell cycle, metabolism, mTOR signaling, PI3K signaling, MAPK signaling, HIF1α signaling, and regulation of cytoskeleton consistent with the proteo-transcriptomic analysis. In addition, we also observed an increase in gene expression associated with DNA repair pathways, ribosome biosynthesis, metabolism, protein biosynthesis, and ECM interactions common to all three treatments. (Fig. 3a). The increase in proliferation, DNA damage repair pathways, and change in the extracellular matrix proteins suggest putative mechanisms for oncogenesis and tumor migration. Association of DEGs with the KEGG enrichment showed mTOR regulated downstream protein synthesis via S6K, EIF4B, nucleotide biosynthesis via ATF4, MTHFD2, S6K, loss of autophagy via FOXO3, ULK1, and cytoskeletal rearrangement via RHO in all three treatments (Fig. S3).

We further analyzed the oncogenesis gene list that was curated previously to investigate the genome-wide expression of oncogenes in all three treatments (Fig. 3c). Increased expression of oncogenes indicated an increase in oncogenic potential consistent with the proteomic analysis. TF enrichment was performed for all DE TFs based and their DE targets and EMT genes as targets from TRANSFAC database. Top TFs were associated with modules such as cell cycle (E2F1, SP3, EGR1), metabolism (NFYA, NFYB), growth, and proliferation (JUN, FOS) (Fig. 3b). EMT target genes were regulated by JUN, TCF7L2, AR, and HIF1A.

To decipher the ligand-specific transcriptional changes, we performed a pairwise comparison analysis using a previously published method (see Methods)[59]. Functional enrichment of ligand-specific DE genes showed regulation of cell cycle, DNA damage repair proteins, PI3K, FOXO, p53 signaling, loss of apoptosis, ECM interaction, focal adhesion, HIPPO signaling, and TGFβ signaling in EGF treatment, and TH17, Osteoclast differentiation, NF-κB, JAK-STAT, IL-17, TNFα signaling, cytokine-receptor interacting and NOD signaling in OSM treatment. We observed TGFβ signaling in EGF and OSM treatments. In EGF treatment EMP3, TGFBR2 RHOA and MAPK1 were upregulated while in OSM treatment, we see upregulation of TGFBR1, SMAD3, ZEB1 and NREP was driven by TGFBR2 activation and its downstream effects and TGFBR1 activation in OSM (Fig. 3f). Treatment-specific TFs and their targets-based gene networks were constructed, and key hub genes such as E2F1, ETS1, and NRF1 were identified (Fig. 3d and e). E2F1 played a key role in EGF specific treatment and led to the regulation of multiple DE targets that participate in cell cycle (CCND1, CDC6, CDC25a, PTTG1, CDK2, PLK1, DFB4, BRCA1, CCNB2, CCNE2) and EMT (SMC2, FGF2, FGFR1, CDKN2C, NRF1). Other hub genes identified in the EGF treatment network were BRCA1, NRF1, THBS1, HMGA1, and FOSL1. These genes contribute to changes in cytoskeletal organization, increased tumor progression, and invasion aiding EMT[60]. OSM treatment showed a repertoire of hub TF such as JUN, FOS, SMAD3, HIF1A, STAT3, CREB1, ESR1, and CEBPB. These TFs regulate EMT, inflammation, and cell cycle. Thus, the transcriptomic analysis shows that E2F1 and its targets were regulated in all three treatments; however, EGF showed enhanced regulation of E2F1 compared to other treatments. AP1 complex (JUN and FOS) were regulated by both E2F1 and STAT3 in the OSM condition, leading to EMT.

**FIG4:**
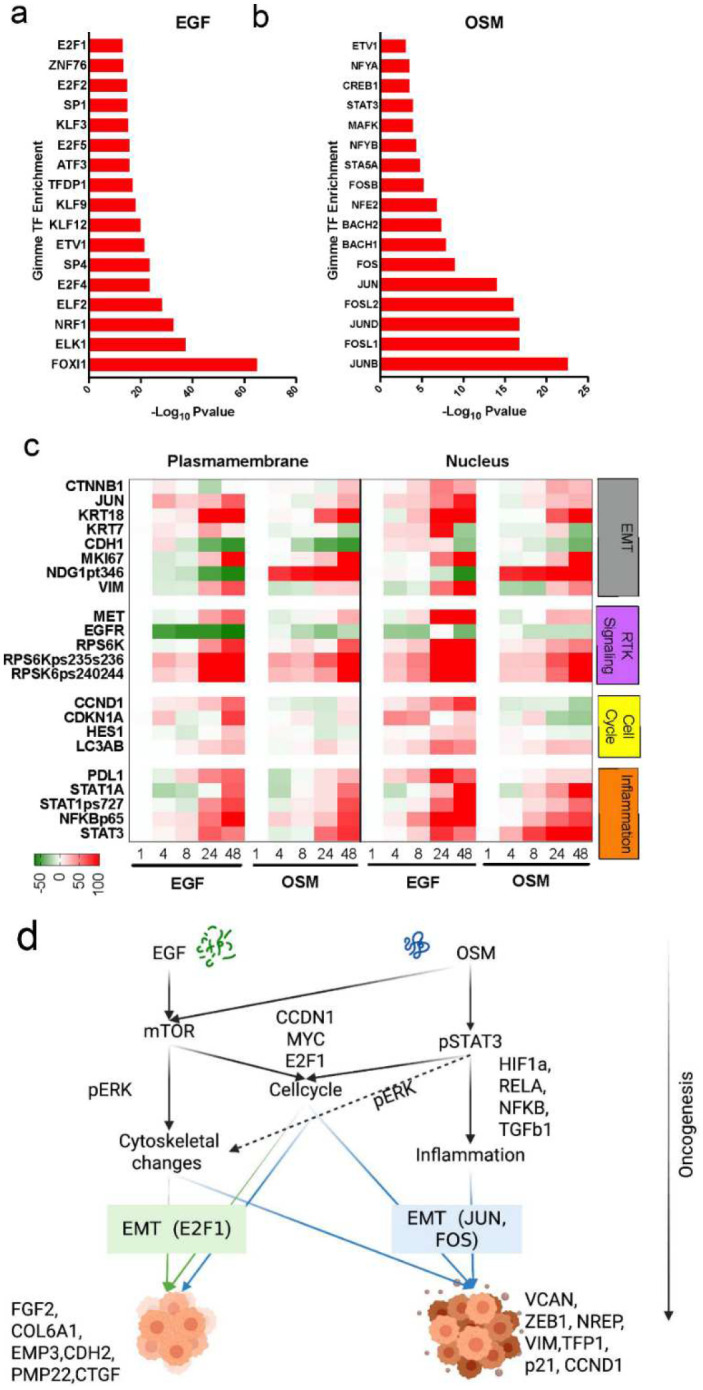
ATAC-seq and Cyclic-IF validation of Oncogenesis and EMT a and b) Gimme motif enrichment analysis of promoter regions using the HOCOMOCOv11database for EGF and OSM treatments respectively at 48 hours. All results presented p-value < 0.05. c)Heat map of Cyclic-IF expression of proteins at 1, 4, 8, 12, 24, and 48 hours annotated into four modules namely EMT, cell cycle, RTK signaling, and inflammation. d)A schematic model of EMT progression in EGF and OSM treatments. EGF and OSM mediated EMT regulating ECM reorganization leading to upregulation of EMT markers (FGF2, COL6A1, PMP22). OSM mediated phosphorylation of STAT3-pT705 leads to activation of canonical TGFβ signaling and concomitant activation of EMT genes (VIM, CCDN1, ZEB1)

Transcriptional changes were validated using changes in chromatin accessibility obtained from the ATAC-seq data. Differentially accessible regions (DARs) were identified using Diffbind[61] in EGF and OSM at 24 and 48 hours with respect to PBS. Peaks occurring in the promoter region [−1000, +500 base pairs (bp) from TSS] were annotated using ChIPseeker[62]. These regions showed enrichment of genes associated with proteoglycans, focal adhesion, RAP signaling, and loss of apoptosis in EGF treatment, and RAP, RAS, and PI3K signaling in OSM treatment. To identify the regulator of these promoter regions, Motif enrichment analysis with GimmeMotifs[63] was performed with the Homer algorithm using the HOCOMOCOv11databbase[64]. In EGF treatment, the analysis revealed binding sites for cell cycle-related TFs and MAPK activator (ELK1),[65, 66] and cell cycle modulators (E2F1, SP2, E2F2)[59, 67–70] (Fig 4a). In OSM treatment, upregulation of differentially accessible regions showed enrichment of AP1 complex (FOSL1, FOSL2, JUNB, JUND, FOS, FOSB), cell cycle, and pro-inflammatory regulators (STAT3, NFYA) (Fig. 4b) These results further supported the role of E2F1 in EGF and AP1 in OSM which were consistent with the transcriptomic analysis.

The cyclic-IF data across time following the treatments shows dynamic changes in localization of the proteins. To associate mechanisms inferred from the omics measurements with phenotypic outcomes, we categorized the cyclic-IF outcomes into EMT, RTK signaling, cell cycle, and inflammation-associated mechanisms. An increase in nuclear and plasma membrane localization of proteins associated with inflammation (STAT1, STAT3, PDL1, NFKB-P65), RTK signaling (MET, RPS6), and EMT proteins (VIM, KRT18, KI67, JUN) were observed in both EGF and OSM treatments (Fig 4c). The EMT progression was supported by the decrease of CDH1 in the plasma membrane and the nucleus and an increase of CTNNB1 in the nucleus in both treatments. The nuclear intensities of NDRG1-pT346 showed an increase in OSM in contrast to EGF treatment. A temporal increase in localization of cell cycle genes (CCND1, CDKN1A, HES1) was seen in EGF. In the OSM treatment, cyclic-IF data showed reduced presence of CCND1, CDKN1A consistent with RNA-seq measurements at 48 h.

## Discussion

The transformation of normal epithelial cells into an oncogenic state is orchestrated by multiple cellular processes. The availability of multi-omics longitudinal measurements on a normal breast epithelial cell treated with distinct growth factors, in the LINCS study, offers the potential to identify key mechanisms. These mechanisms poise the epithelial cell for transition to a tumor, and through analyses of the LINCS measurements we identify the pathways that are pro-oncogenic and transformation-resistant and explore temporal causality of these mechanisms[71, 72]. EGF is known to play a key role in promoting cell proliferation, differentiation, and survival[73], while HGF, which is largely similar in response, additionally regulates cell migration mechanisms[74]. OSM, another native epithelial cell factor, plays a key role in controlling proliferation while increasing cell motility[75]. Our analyses of LINCS MCF10a data broadly support these mechanisms while providing significant detailed insights into phenotypic changes that promote tumorigenic transformation of epithelial cells.

All three treatments demonstrate changes in cell state that contribute to pro and anti-oncogenic cell fates. EGF, HGF and OSM, regulate mTOR, AKT and ERK pathways which promote oncogenic transformation in epithelial cells[76–78]. Notably the expression of oncogenes and oncoproteins such as BRCA1, BRCA2, CCND1, CCNE1, HER2, EGFR, c-KIT, MET, TP53, ATM, CHEK1, CHEK2, NF1, PDL1, have been reported to be elevated in breast cancer cells and associated with increased cell cycle, metabolism, DNA damage repair pathways and EMT[79]. Consistent with these findings, most breast cancer genes are upregulated in all three treatments, according to our study. It has been shown that mTOR signaling regulates proliferation via EIF4EBP1 and S6K, metabolism and immune response via AKT, NDRG1, PDK1 and PRKCA[80]. This regulation of downstream targets of mTOR is also seen with EGF and HGF and OSM treatments[81–84] leading to increased proliferation and EMT. In addition, OSM, as an analog of IL6, regulates cytokine signaling and shows the activation of immune response[85]. Thus, these growth regulators play a pivotal role during initial stages of cell states that lead to oncogenic transformation.

Our analysis of the multi-omics measurements of response to ligand treatments provides unique mechanistic insights. We identified various pathways in which EGF regulated oncogenesis. In our analysis, E2F1 is seen to be regulated by EGF through NRF1, RB1, and mTOR signaling and leads to increase in both cell cycle and EMT. This is consistent with CDK-RB-E2F pathway that is critical for the control of cell proliferation, angiogenesis, metastasis, and tumor progression[86]. EGF is also shown to transactivate TGFβ receptor signaling that leads to EMT[87]. Further, E2F1 can lead to transcriptional activation of ZEB1 which causes cytoskeletal remodeling by binding to the proximal promoter region and recruiting HDAC1/2, while also downregulating CDH1. This relation between E2F1, ZEB1 and CDH1 is also observed in our data. In addition to E2F1 regulation through mTOR, OSM treatment also shows activation of JAK/STAT signaling leading to enhanced EMT. Increase in EMT is also supported by the temporal activation of STAT3-pY705 as seen in RPPA, increased TF activity in transcriptomic and nuclear localization in cyclic-IF measurements[88–90]. We identified two key downstream effects due to STAT3 signaling namely activation of AP1 complex and regulation of hypoxia via HIF1A. AP1 binds in the promoter region and leads an increase in FN1, VIM and CDH2[91, 92] AP1 also regulates ZEB1 and SNAI1, which are known EMT TFs [93, 94] Transcriptional regulation of HIF1A led to the regulation of EMT (PLAUR, CXCL8, FGF2) and angiogenesis (VEGFA, ITGB1, PTGS2). The relationship between HIF1A-induced hypoxia and OSM treatment can be attributed to metabolic changes and molecular changes consistent with EMT in epithelial cells.[95, 96]

EGF and OSM treatments showed distinct EMT responses. EGF treatment shows EMT is regulated by E2F1. It is accompanied by an increase in the expression of CTGF, NR2F1, CDH2, PMP22, CDKN2C, FGF2, and EMP3 showing an upregulation of the cell-cycle. Under OSM treatment, EMT is regulated by E2F1 and AP1. The downstream signaling indicates that STAT3 drives pro-inflammatory cytokine signaling. Further, extracellular matrix organization (VCAN, VIM, ADAM12, LIM, FBN1, LTBP2), inflammatory factors (TNFAIP6) TGFβ pathway (TFP1, NREP) and enzyme activity (SULF1, TGM2) are regulated in OSM treatment. Differences in the EMT signaling can be attributed to the differences in the signaling of TGFβ receptors[97]. It is shown in literature that EGF activates the TGFβ receptor and, OSM treatment activates the TGFβ via STAT3 which causes canonical TGFβ signaling via SMAD activation[98].

## Conclusion:

Taken together, our analyses of the LINCS measurements provide an integrated multi-omics insight into initiation and progression of oncogenesis. All three growth regulators, EGF, HGF, and OSM, activated downstream pathways mTOR, ERK, and MAPK leading to increased pro-oncogenic processes, and EMT. The TF-target analysis identified the role of E2F1 mediated oncogenesis and EMT in all the ligands. In addition to the role of E2F1, in OSM treatment, STAT3 led AP1 activity led to a strong EMT signal. This suggests potential mechanisms for growth factor-driven oncogenesis. Targeting these mechanisms has the potential to interfere with pro-oncogenic mechanisms and alter pathological cell states. In vivo, several other cell-intrinsic growth factors and cytokines crosstalk participate in cell fate decisions. To explore the combinatorial effects of such stimuli, we study in a companion paper the effect of endogenous ligands namely TGFβ, BMP2, and IFNγ in combination with EGF in determining the pro- and anti-oncogenic cell fates of normal breast epithelial cells.

## Supplementary Material

Supplementary Files

This is a list of supplementary files associated with this preprint. Click to download.

- Supplementary.docx

## Figures and Tables

**Figure Fig1:**
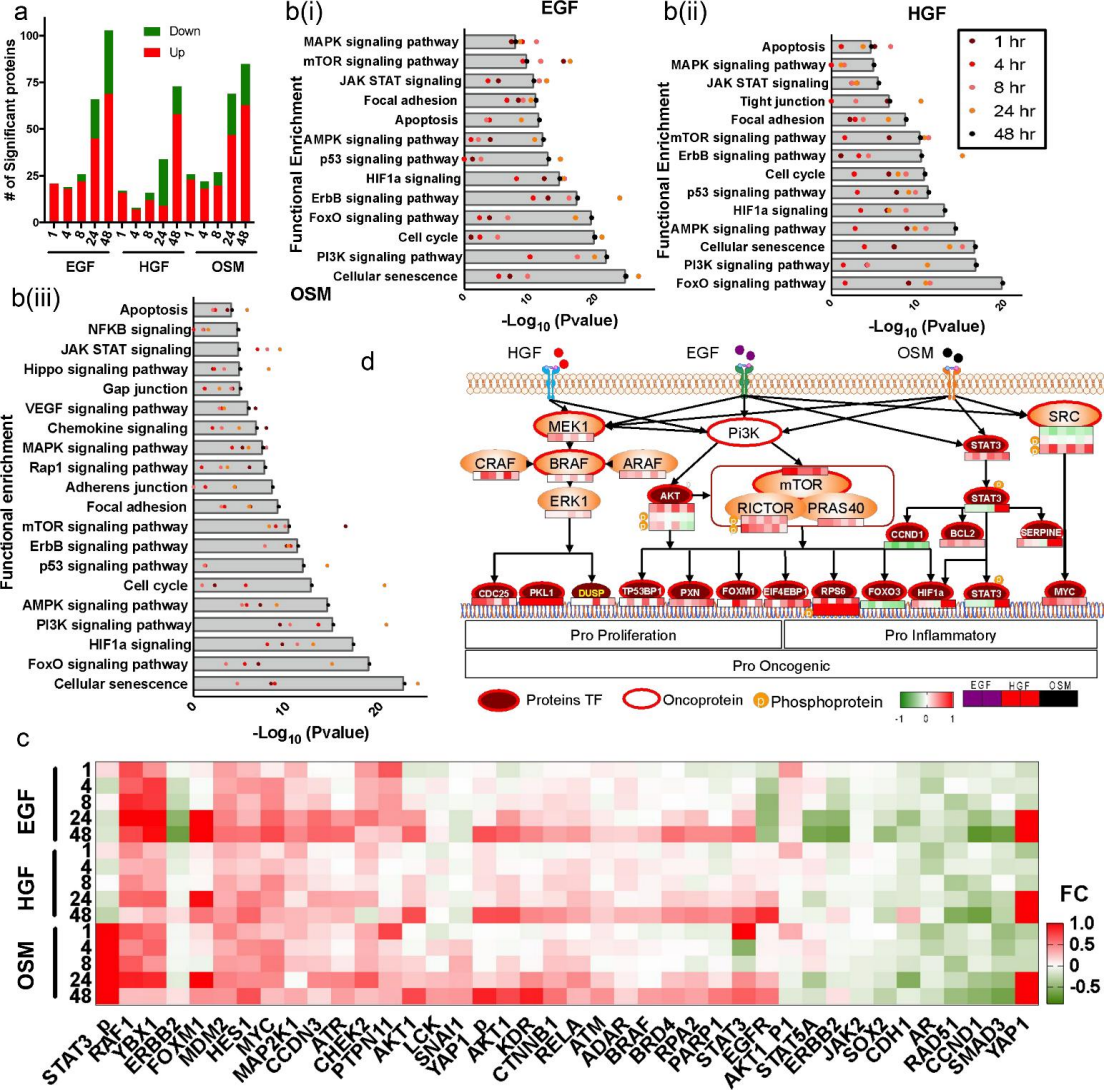
Role of RPPA proteins in the initiation of oncogenic process. a) Bar plots of number of up and down-regulated proteins from RPPA measurement in EGF, HGF, and OSM treatments compared to PBS control at 1, 4, 8, 24, and 48 hours show a temporal increase in significant proteins. Green represents number of downregulated and red represents number of upregulated proteins. b) Dot plot of cellular signaling pathway enrichment of significant proteins in (i)EGF, (ii)HGF, and (iii) OSM treatment; functional enrichment at each time points plotted vs −log_10_(p-value). Dots represent the time points 1, 4, 8, 24, and 48 hours. The bar represents the −log_10_(p-value) at 48 hours. c) Fold change heatmap of significant RPPA oncogenic proteins at 1, 4, 8, 24, and 48 hours respectively, shows an increase in expression of oncogenic proteins. d) A schematic model of the protein signaling response to EGF, HGF, and OSM treatments along with the FC heatmaps for the significant proteins at 24- and 48-hour time points (EGF, HGF, OSM are represented pairwise in sequence). All treatments signal via oncogenic pathways namely mTOR, ERK, RAF leading to an increase in proliferation by regulation of CDC25, TP53BP1, PXN, EIF4EBP1, RPS6, FOXM1 TFs. In addition, OSM signals via oncogenic activation of STAT3 signaling and leads to an increase in inflammation through HIF1A and STAT3 TFs. Data of phosphoproteins (p) is represented as an additional row.

## Data Availability

Data was downloaded from website: synapse.org/LINCS_MCF10A

## Abbreviations:

LINCS: Library of Integrated Network-based Cellular Signatures
RPPA: Reverse phase protein Assay
Cyclic-IF: Cyclic immune-fluorescence
EMT: Epithelial to Mesenchymal Transition
OSM: Oncostatin-M

## References

[1] Andor N, Graham TA, Aktipis AC, Petritsch C, Ji HP, Maley CC. Pan-cancer analysis of the causes and consequences of Intra-tumor heterogeneity. Cancer Research. 2015;75.

[2] Marusyk A, Polyak K. Tumor heterogeneity: Causes and consequences. Biochimica Et Biophysica Acta-Reviews on Cancer. 2010;1805:105–117. 10.1016/j.bbcan.2009.11.002 PMC2814927 19931353

[3] Oh BY, Shin HT, Yun JW, Intratumor heterogeneity inferred from targeted deep sequencing as a prognostic indicator. Scientific Reports. 2019;9. 10.1038/s41598-019-41098-0 PMC6418103 30872730

[4] Perou CM, Sørlie T, Eisen MB, Molecular portraits of human breast tumours. Nature. 2000;406:747–752. 10963602 10.1038/35021093

[5] Rybinski B, Yun K. Addressing intra-tumoral heterogeneity and therapy resistance. Oncotarget. 2016;7:72322–72342. 27608848 10.18632/oncotarget.11875 PMC5342165

[6] Lim ZF, Ma PC. Emerging insights of tumor heterogeneity and drug resistance mechanisms in lung cancer targeted therapy. Journal of Hematology & Oncology. 2019;12. 10.1186/s13045-019-0818-2 PMC6902404 31815659

[7] Mercurio AM, Bachelder RE, Bates RC, Chung J. Autocrine signaling in carcinoma: VEGF and the alpha 6 beta 4 integrin. Seminars in Cancer Biology. 2004;14:115–122. 15018895 10.1016/j.semcancer.2003.09.016

[8] Lokker NA, Sullivan CM, Hollenbach SJ, Israel MA, Giese NA. Platelet-derived growth factor (PDGF) autocrine signaling regulates survival and mitogenic pathways in glioblastoma cells: Evidence that the novel PDGF-C and PDGF-D ligands may play a role in the development of brain tumors. Cancer Research. 2002;62:3729–3735. 12097282

[9] Papageorgis P, Stylianopoulos T. Role of TGF beta in regulation of the tumor microenvironment and drug delivery. International Journal of Oncology. 2015;46:933–943. 25573346 10.3892/ijo.2015.2816 PMC4306018

[10] Nickerson NK, Mill CP, Wu HJ, Riese DJ, Foley J. Autocrine-Derived Epidermal Growth Factor Receptor Ligands Contribute to Recruitment of Tumor-Associated Macrophage and Growth of Basal Breast Cancer Cells In Vivo. Oncology Research. 2012;20:303–317. 10.3727/096504013x13639794277761 PMC3984049 23879171

[11] COHEN S, LEWIS HB. The nitrogenous metabolism of the earthworm (Lumbricus terrestric). II. Arginase and urea synthesis. J Biol Chem. 1950;184:479–484. 15428427

[12] Owusu BY, Galemmo R, Janetka J, Klampfer L. Hepatocyte Growth Factor, a Key Tumor-Promoting Factor in the Tumor Microenvironment. Cancers. 2017;9. 10.3390/cancers9040035 PMC5406710 28420162

[13] Imamura T, Hikita A, Inoue Y. The roles of TGF-beta signaling in carcinogenesis and breast cancer metastasis. Breast Cancer. 2012;19:118–124. 22139728 10.1007/s12282-011-0321-2

[14] Padua D, Massague J. Roles of TGF beta in metastasis. Cell Research. 2009;19:89–102. 19050696 10.1038/cr.2008.316

[15] Shanmugalingam T, Bosco C, Ridley AJ, Van Hemelrijck M. Is there a role for IGF-1 in the development of second primary cancers? Cancer Medicine. 2016;5:3353–3367. 27734632 10.1002/cam4.871 PMC5119990

[16] West NR, Murray JI, Watson PH. Oncostatin-M promotes phenotypic changes associated with mesenchymal and stem cell-like differentiation in breast cancer. Oncogene. 2014;33:1485–1494. 23584474 10.1038/onc.2013.105

[17] McFarland JM, Ho ZV, Kugener G, Improved estimation of cancer dependencies from large-scale RNAi screens using model-based normalization and data integration. Nature Communications. 2018;9. 10.1038/s41467-018-06916-5 PMC6214982 30389920

[18] Doherty MR, Parvani JG, Tamagno I, The opposing effects of interferon-beta and oncostatin-M as regulators of cancer stem cell plasticity in triple-negative breast cancer. Breast Cancer Res. 2019;21:54. 31036052 10.1186/s13058-019-1136-x PMC6489282

[19] Mullenders J, Bernards R. Loss-of-function genetic screens as a tool to improve the diagnosis and treatment of cancer. Oncogene. 2009;28:4409–4420. 19767776 10.1038/onc.2009.295

[20] Hart T, Chandrashekhar M, Aregger M, High-Resolution CRISPR Screens Reveal Fitness Genes and Genotype-Specific Cancer Liabilities. Cell. 2015;163. 10.1016/j.cell.2015.11.015 26627737

[21] Holbro T, Beerli RR, Maurer F, Koziczak M, Barbas CF, Hynes NE. The ErbB2/ErbB3 heterodimer functions as an oncogenic unit: ErbB2 requires ErbB3 to drive breast tumor cell proliferation. Proceedings of the National Academy of Sciences of the United States of America. 2003;100:8933–8938. 12853564 10.1073/pnas.1537685100 PMC166416

[22] Faltus T, Yuan JP, Zimmer B, Silencing of the HER2/neu gene by siRNA inhibits proliferation and induces apoptosis in HER2/neu-overexpressing breast cancer cells. Neoplasia. 2004;6:786–795. 15720805 10.1593/neo.04313 PMC1531682

[23] Camerini A, Amoroso D, Capodanno A, Correlation of cyclin E expression with manganese superoxide dismutase expression and prediction of survival in early breast cancer patients receiving epirubicin-based adjuvant chemotherapy. Journal of Clinical Oncology. 2008;26.

[24] Tan AR, Swain SM. Review of flavopiridol, a cyclin-dependent kinase inhibitor, as breast cancer therapy. Seminars in Oncology. 2002;29:77–85. 10.1053/sonc.2002.34059 12138401

[25] Andreopoulou E, Sparano JA. Chemotherapy in Patients with Anthracycline and Taxane-Pretreated Metastatic Breast Cancer: An Overview. Current Breast Cancer Reports. 2013;5:42–50. 23440080 10.1007/s12609-012-0097-1 PMC3579672

[26] D’Alesio C, Punzi S, Cicalese A, RNAi screens identify CHD4 as an essential gene in breast cancer growth. Oncotarget. 2016;7:80901–80915. 27779108 10.18632/oncotarget.12646 PMC5348363

[27] Yuan TL, Cantley LC. PI3K pathway alterations in cancer: variations on a theme. Oncogene. 2008;27:5497–5510. 18794884 10.1038/onc.2008.245 PMC3398461

[28] Li L, Feng Y, Hu S, ZEB1 serves as an oncogene in acute myeloid leukaemia via regulating the PTEN/PI3K/AKT signalling pathway by combining with P53. J Cell Mol Med. 2021;25:5295–5304. 33960640 10.1111/jcmm.16539 PMC8178252

[29] Chun J, Song K, Kim YS. Sesquiterpene lactones-enriched fraction of Inula helenium L. induces apoptosis through inhibition of signal transducers and activators of transcription 3 signaling pathway in MDA-MB-231 breast cancer cells. Phytother Res. 2018;32:2501–2509. 30251272 10.1002/ptr.6189

[30] Xie Q, Yang Z, Huang X, Ilamycin C induces apoptosis and inhibits migration and invasion in triple-negative breast cancer by suppressing IL-6/STAT3 pathway. J Hematol Oncol. 2019;12:60. 31186039 10.1186/s13045-019-0744-3 PMC6558915

[31] Tolomeo M, Cascio A. The Multifaced Role of STAT3 in Cancer and Its Implication for Anticancer Therapy. Int J Mol Sci. 2021;22. 10.3390/ijms22020603 PMC7826746 33435349

[32] Kallioniemi OP, Wagner U, Kononen J, Sauter G. Tissue microarray technology for high-throughput molecular profiling of cancer. Human Molecular Genetics. 2001;10:657–662. 11257096 10.1093/hmg/10.7.657

[33] Ghandi M, Huang FW, Jane-Valbuena J, Next-generation characterization of the Cancer Cell Line Encyclopedia. Nature. 2019;569:503−+. 31068700 10.1038/s41586-019-1186-3 PMC6697103

[34] Granja JM, Klemm S, McGinnis LM, Single-cell multiomic analysis identifies regulatory programs in mixed-phenotype acute leukemia. Nature Biotechnology. 2019;37:1458−+. 10.1038/s41587-019-0332-7 PMC7258684 31792411

[35] Charoentong P, Finotello F, Angelova M, Pan-cancer Immunogenomic Analyses Reveal Genotype-Immunophenotype Relationships and Predictors of Response to Checkpoint Blockade. Cell Reports. 2017;18:248–262. 28052254 10.1016/j.celrep.2016.12.019

[36] de Anda-Jauregui G, Hernandez-Lemus E. Computational Oncology in the Multi-Omics Era: State of the Art. Frontiers in Oncology. 2020;10. 10.3389/fonc.2020.00423 PMC7154096 32318338

[37] Nicora G, Vitali F, Dagliati A, Geifman N, Bellazzi R. Integrated Multi-Omics Analyses in Oncology: A Review of Machine Learning Methods and Tools. Frontiers in Oncology. 2020;10. 10.3389/fonc.2020.01030 PMC7338582 32695678

[38] Menyhart O, Gyorffy B. Multi-omics approaches in cancer research with applications in tumor subtyping, prognosis, and diagnosis. Comput Struct Biotechnol J. 2021;19:949–960. 33613862 10.1016/j.csbj.2021.01.009 PMC7868685

[39] Gross SM, Dane MA, Smith RL, A multi-omic analysis of MCF10A cells provides a resource for integrative assessment of ligand-mediated molecular and phenotypic responses. Commun Biol. 2022;5:1066. 36207580 10.1038/s42003-022-03975-9 PMC9546880

[40] Keenan AB, Jenkins SL, Jagodnik KM, The Library of Integrated Network-Based Cellular Signatures NIH Program: System-Level Cataloging of Human Cells Response to Perturbations. Cell Syst. 2018;6:13–24. 29199020 10.1016/j.cels.2017.11.001 PMC5799026

[41] Lee EY, Muller WJ. Oncogenes and tumor suppressor genes. Cold Spring Harb Perspect Biol. 2010;2:a003236. 20719876 10.1101/cshperspect.a003236 PMC2944361

[42] Sjoblom T, Jones S, Wood LD, The consensus coding sequences of human breast and colorectal cancers. Science. 2006;314:268–274. 16959974 10.1126/science.1133427

[43] Zhu K, Liu Q, Zhou Y, Oncogenes and tumor suppressor genes: comparative genomics and network perspectives. BMC Genomics. 2015;16 Suppl 7:S8. 10.1186/1471-2164-16-S7-S8 PMC4474543 26099335

[44] Kudo-Saito C, Shirako H, Takeuchi T, Kawakami Y. Cancer metastasis is accelerated through immunosuppression during Snail-induced EMT of cancer cells. Cancer Cell. 2009;15:195–206. 19249678 10.1016/j.ccr.2009.01.023

[45] Burk U, Schubert J, Wellner U, A reciprocal repression between ZEB1 and members of the miR-200 family promotes EMT and invasion in cancer cells. EMBO Rep. 2008;9:582–589. 18483486 10.1038/embor.2008.74 PMC2396950

[46] Fedele M, Cerchia L, Chiappetta G. The Epithelial-to-Mesenchymal Transition in Breast Cancer: Focus on Basal-Like Carcinomas. Cancers (Basel). 2017;9. 10.3390/cancers9100134 PMC5664073 28974015

[47] Ye X, Brabletz T, Kang Y, Upholding a role for EMT in breast cancer metastasis. Nature. 2017;547:E1–E3. 28682326 10.1038/nature22816 PMC6283276

[48] Liu F, Gu LN, Shan BE, Geng CZ, Sang MX. Biomarkers for EMT and MET in breast cancer: An update. Oncol Lett. 2016;12:4869–4876. 28105194 10.3892/ol.2016.5369 PMC5228449

[49] Wu HT, Zhong HT, Li GW, Oncogenic functions of the EMT-related transcription factor ZEB1 in breast cancer. J Transl Med. 2020;18:51. 32014049 10.1186/s12967-020-02240-z PMC6998212

[50] Zhao M, Liu Y, Qu H. Expression of epithelial-mesenchymal transition-related genes increases with copy number in multiple cancer types. Oncotarget. 2016;7:24688–24699. 27029057 10.18632/oncotarget.8371 PMC5029734

[51] Peixoto P, Etcheverry A, Aubry M, EMT is associated with an epigenetic signature of ECM remodeling genes. Cell Death Dis. 2019;10:205. 30814494 10.1038/s41419-019-1397-4 PMC6393505

[52] Ariazi EA, Taylor JC, Black MA, A New Role for ERα: Silencing via DNA Methylation of Basal, Stem Cell, and EMT Genes. Mol Cancer Res. 2017;15:152–164. 28108626 10.1158/1541-7786.MCR-16-0283 PMC5308451

[53] Li Z, Wang Y, Kong L, Yue Z, Ma Y, Chen X. Expression of ADAM12 is regulated by E2F1 in small cell lung cancer. Oncol Rep. 2015;34:3231–3237. 26503019 10.3892/or.2015.4317

[54] Smigiel JM, Parameswaran N, Jackson MW. Potent EMT and CSC Phenotypes Are Induced By Oncostatin-M in Pancreatic Cancer. Mol Cancer Res. 2017;15:478–488. 28053127 10.1158/1541-7786.MCR-16-0337 PMC5380554

[55] Pollack V, Sarkozi R, Banki Z, Oncostatin M-induced effects on EMT in human proximal tubular cells: differential role of ERK signaling. Am J Physiol Renal Physiol. 2007;293:F1714–1726. 17881458 10.1152/ajprenal.00130.2007

[56] Dinca SC, Greiner D, Weidenfeld K, Bond L, Barkan D, Jorcyk CL. Novel mechanism for OSM-promoted extracellular matrix remodeling in breast cancer: LOXL2 upregulation and subsequent ECM alignment. Breast Cancer Res. 2021;23:56. 34011405 10.1186/s13058-021-01430-x PMC8132418

[57] De Las Rivas J, Brozovic A, Izraely S, Casas-Pais A, Witz IP, Figueroa A. Cancer drug resistance induced by EMT: novel therapeutic strategies. Arch Toxicol. 2021;95:2279–2297. 34003341 10.1007/s00204-021-03063-7 PMC8241801

[58] Caldon CE, Daly RJ, Sutherland RL, Musgrove EA. Cell cycle control in breast cancer cells. J Cell Biochem. 2006;97:261–274. 16267837 10.1002/jcb.20690

[59] Zhang W, Shi X, Peng Y, HIF-1α Promotes Epithelial-Mesenchymal Transition and Metastasis through Direct Regulation of ZEB1 in Colorectal Cancer. PLoS One. 2015;10:e0129603. 26057751 10.1371/journal.pone.0129603 PMC4461314

[60] Wu DY, Bittencourt D, Stallcup MR, Siegmund KD. Identifying differential transcription factor binding in ChIP-seq. Front Genet. 2015;6:169. 25972895 10.3389/fgene.2015.00169 PMC4413818

[61] Wang Q, Li M, Wu T, Exploring Epigenomic Datasets by ChIPseeker. Curr Protoc. 2022;2:e585. 36286622 10.1002/cpz1.585

[62] van Heeringen SJ, Veenstra GJ. GimmeMotifs: a de novo motif prediction pipeline for ChIP-sequencing experiments. Bioinformatics. 2011;27:270–271. 21081511 10.1093/bioinformatics/btq636 PMC3018809

[63] Kulakovskiy IV, Vorontsov IE, Yevshin IS, HOCOMOCO: towards a complete collection of transcription factor binding models for human and mouse via large-scale ChIP-Seq analysis. Nucleic Acids Res. 2018;46:D252–D259. 29140464 10.1093/nar/gkx1106 PMC5753240

[64] Rao VN, Reddy ES. elk-1 proteins interact with MAP kinases. Oncogene. 1994;9:1855–1860. 8208531

[65] Marin-Kuan M, Nestler S, Verguet C, MAPK-ERK activation in kidney of male rats chronically fed ochratoxin A at a dose causing a significant incidence of renal carcinoma. Toxicol Appl Pharmacol. 2007;224:174–181. 17651772 10.1016/j.taap.2007.06.014

[66] Senderowicz AM. Cell cycle modulators for the treatment of lung malignancies. Clin Lung Cancer. 2003;5:158–168. 14667271 10.3816/CLC.2003.n.028

[67] Guarducci C, Bonechi M, Benelli M, Cyclin E1 and Rb modulation as common events at time of resistance to palbociclib in hormone receptor-positive breast cancer. NPJ Breast Cancer. 2018;4:38. 30511015 10.1038/s41523-018-0092-4 PMC6261939

[68] Schwartz GK, Shah MA. Targeting the cell cycle: a new approach to cancer therapy. J Clin Oncol. 2005;23:9408–9421. 16361640 10.1200/JCO.2005.01.5594

[69] Fernandez PL, Jares P, Rey MJ, Campo E, Cardesa A. Cell cycle regulators and their abnormalities in breast cancer. Mol Pathol. 1998;51:305–309. 10193510 10.1136/mp.51.6.305 PMC395656

[70] Witsch E, Sela M, Yarden Y. Roles for growth factors in cancer progression. Physiology (Bethesda). 2010;25:85–101. 20430953 10.1152/physiol.00045.2009 PMC3062054

[71] Zandi R, Larsen AB, Andersen P, Stockhausen MT, Poulsen HS. Mechanisms for oncogenic activation of the epidermal growth factor receptor. Cell Signal. 2007;19:2013–2023. 17681753 10.1016/j.cellsig.2007.06.023

[72] Qu Y, Han B, Yu Y, Evaluation of MCF10A as a Reliable Model for Normal Human Mammary Epithelial Cells. PLoS One. 2015;10:e0131285. 26147507 10.1371/journal.pone.0131285 PMC4493126

[73] Leroy C, Deheuninck J, Reveneau S, HGF/SF regulates expression of apoptotic genes in MCF-10A human mammary epithelial cells. Ann N Y Acad Sci. 2006;1090:188–202. 17384262 10.1196/annals.1378.021

[74] Tiffen PG, Omidvar N, Marquez-Almuina N, Croston D, Watson CJ, Clarkson RW. A dual role for oncostatin M signaling in the differentiation and death of mammary epithelial cells in vivo. Mol Endocrinol. 2008;22:2677–2688. 18927239 10.1210/me.2008-0097 PMC5419408

[75] Galbaugh T, Cerrito MG, Jose CC, Cutler ML. EGF-induced activation of Akt results in mTOR-dependent p70S6 kinase phosphorylation and inhibition of HC11 cell lactogenic differentiation. BMC Cell Biol. 2006;7:34. 16984645 10.1186/1471-2121-7-34 PMC1590014

[76] Graupera M, Potente M. Regulation of angiogenesis by PI3K signaling networks. Exp Cell Res. 2013;319:1348–1355. 23500680 10.1016/j.yexcr.2013.02.021

[77] Hermanns HM. Oncostatin M and interleukin-31: Cytokines, receptors, signal transduction and physiology. Cytokine Growth Factor Rev. 2015;26:545–558. 26198770 10.1016/j.cytogfr.2015.07.006

[78] Osborne C, Wilson P, Tripathy D. Oncogenes and tumor suppressor genes in breast cancer: potential diagnostic and therapeutic applications. Oncologist. 2004;9:361–377. 15266090 10.1634/theoncologist.9-4-361

[79] Kim LC, Cook RS, Chen J. mTORC1 and mTORC2 in cancer and the tumor microenvironment. Oncogene. 2017;36:2191–2201. 27748764 10.1038/onc.2016.363 PMC5393956

[80] Chen KS, Fustino NJ, Shukla AA, EGF Receptor and mTORC1 Are Novel Therapeutic Targets in Nonseminomatous Germ Cell Tumors. Mol Cancer Ther. 2018;17:1079–1089. 29483210 10.1158/1535-7163.MCT-17-0137 PMC5932259

[81] Shrivastava R, Asif M, Singh V, M2 polarization of macrophages by Oncostatin M in hypoxic tumor microenvironment is mediated by mTORC2 and promotes tumor growth and metastasis. Cytokine. 2019;118:130–143. 29625858 10.1016/j.cyto.2018.03.032

[82] Houben E, Hellings N, Broux B. Oncostatin M, an Underestimated Player in the Central Nervous System. Front Immunol. 2019;10:1165. 31191538 10.3389/fimmu.2019.01165 PMC6549448

[83] Xie J, Proud CG. Signaling crosstalk between the mTOR complexes. Translation (Austin). 2014;2:e28174. 26779402 10.4161/trla.28174 PMC4705829

[84] Dey G, Radhakrishnan A, Syed N, Signaling network of Oncostatin M pathway. J Cell Commun Signal. 2013;7:103–108. 23255051 10.1007/s12079-012-0186-y PMC3660694

[85] Johnson J, Thijssen B, McDermott U, Garnett M, Wessels LF, Bernards R. Targeting the RB-E2F pathway in breast cancer. Oncogene. 2016;35:4829–4835. 26923330 10.1038/onc.2016.32 PMC4950965

[86] Liu R, Zhang Y, Ding Y, Zhang S, Pan L. Characteristics of TGFBR1-EGFR-CTNNB1-CDH1 Signaling Axis in Wnt-Regulated Invasion and Migration in Lung Cancer. Cell Transplant. 2020;29:963689720969167. 10.1177/0963689720969167 PMC7784602 33231090

[87] Lin WH, Chang YW, Hong MX, STAT3 phosphorylation at Ser727 and Tyr705 differentially regulates the EMT-MET switch and cancer metastasis. Oncogene. 2021;40:791–805. 33262462 10.1038/s41388-020-01566-8 PMC7843420

[88] Khan FM, Marquardt S, Gupta SK, Unraveling a tumor type-specific regulatory core underlying E2F1-mediated epithelial-mesenchymal transition to predict receptor protein signatures. Nat Commun. 2017;8:198. 28775339 10.1038/s41467-017-00268-2 PMC5543083

[89] Hollern DP, Swiatnicki MR, Rennhack JP, E2F1 Drives Breast Cancer Metastasis by Regulating the Target Gene FGF13 and Altering Cell Migration. Sci Rep. 2019;9:10718. 31341204 10.1038/s41598-019-47218-0 PMC6656723

[90] Zeng S, Xie X, Xiao YF, Long noncoding RNA LINC00675 enhances phosphorylation of vimentin on Ser83 to suppress gastric cancer progression. Cancer Lett. 2018;412:179–187. 29107103 10.1016/j.canlet.2017.10.026

[91] Chatterjee K, Jana S, DasMahapatra P, Swarnakar S. EGFR-mediated matrix metalloproteinase-7 up-regulation promotes epithelial-mesenchymal transition via ERK1-AP1 axis during ovarian endometriosis progression. FASEB J. 2018;32:4560–4572. 29558202 10.1096/fj.201701382RR

[92] Hsiao YJ, Su KY, Hsu YC, SPANXA suppresses EMT by inhibiting c-JUN/SNAI2 signaling in lung adenocarcinoma. Oncotarget. 2016;7:44417–44429. 27323831 10.18632/oncotarget.10088 PMC5190107

[93] Feldker N, Ferrazzi F, Schuhwerk H, Genome-wide cooperation of EMT transcription factor ZEB1 with YAP and AP-1 in breast cancer. EMBO J. 2020;39:e103209. 32692442 10.15252/embj.2019103209 PMC7459422

[94] Battello N, Zimmer AD, Goebel C, The role of HIF-1 in oncostatin M-dependent metabolic reprogramming of hepatic cells. Cancer Metab. 2016;4:3. 26889381 10.1186/s40170-016-0141-0 PMC4756539

[95] Daly CS, Flemban A, Shafei M, Conway ME, Qualtrough D, Dean SJ. Hypoxia modulates the stem cell population and induces EMT in the MCF-10A breast epithelial cell line. Oncol Rep. 2018;39:483–490. 29207201 10.3892/or.2017.6125 PMC5783614

[96] Tretbar S, Krausbeck P, MUller A, TGF-β inducible epithelial-to-mesenchymal transition in renal cell carcinoma. Oncotarget. 2019;10:1507–1524. 30863498 10.18632/oncotarget.26682 PMC6407676

[97] Bryson BL, Junk DJ, Cipriano R, Jackson MW. STAT3-mediated SMAD3 activation underlies Oncostatin M-induced Senescence. Cell Cycle. 2017;16:319–334. 27892764 10.1080/15384101.2016.1259037 PMC5324753

[98] Love MI, Huber W, Anders S. Moderated estimation of fold change and dispersion for RNA-seq data with DESeq2. Genome Biol. 2014;15:550. 25516281 10.1186/s13059-014-0550-8 PMC4302049

